# Co-Chaperone HSJ1a Dually Regulates the Proteasomal Degradation of Ataxin-3

**DOI:** 10.1371/journal.pone.0019763

**Published:** 2011-05-19

**Authors:** Xue-Chao Gao, Chen-Jie Zhou, Zi-Ren Zhou, Yu-Hang Zhang, Xue-Ming Zheng, Ai-Xin Song, Hong-Yu Hu

**Affiliations:** State Key Laboratory of Molecular Biology, Institute of Biochemistry and Cell Biology, Shanghai Institutes for Biological Sciences, Chinese Academy of Sciences, Shanghai, China; University of Pittsburg, United States of America

## Abstract

Homo sapiens J domain protein (HSJ1) is a J-domain containing co-chaperone that is known to stimulate ATPase activity of HSP70 chaperone, while it also harbors two ubiquitin (Ub)-interacting motifs (UIMs) that may bind with ubiquitinated substrates and potentially function in protein degradation. We studied the effects of HSJ1a on the protein levels of both normal and the disease–related polyQ-expanded forms of ataxin-3 (Atx3) in cells. The results demonstrate that the N-terminal J-domain and the C-terminal UIM domain of HSJ1a exert opposite functions in regulating the protein level of cellular overexpressed Atx3. This dual regulation is dependent on the binding of the J-domain with HSP70, and the UIM domain with polyUb chains. The J-domain down-regulates the protein level of Atx3 through HSP70 mediated proteasomal degradation, while the UIM domain may alleviate this process via maintaining the ubiquitinated Atx3. We propose that co-chaperone HSJ1a orchestrates the balance of substrates in stressed cells in a *Yin-Yang* manner.

## Introduction

The homeostasis of proteins is vital to the physiological activities and health of eukaryotic cells [1], [2], which is influenced by many intrinsic and environmental factors. Besides synthesis, protein folding and degradation are the two major post-translational factors to maintain this particular process [3]. However, many reasons endogenously or exogenously, such as gene mutation, protein overexpression and dislocation, or chemical stresses, can cause protein misfolding or aberrant degradation [4], [5], [6]. For a protein, the failure to refold to its native state or to be eliminated for recycling often leads to protein aggregation, dysfunction, and ultimately cell death, which is implicated in many neurodegenerative diseases, such as Alzheimer's disease, Huntington's disease and Spinocerebellar ataxias [5], [6], [7]. A histopathological hallmark of these diseases is that the misfolded proteins form neuronal intracellular inclusions. Many pathological polyglutamine (polyQ) containing inclusions have been found to be co-localized with HSP70 and its co-chaperones, ubiquitin (Ub) and proteasome subunits [8], [9], [10], [11]. Overexpression of HSP70 and its co-chaperone can reduce inclusion formation and suppress the cell death [12], [13]. These findings suggest that molecular chaperones and the Ub-proteasome system (UPS) are contributable to refold or eliminate the misfolded proteins before their aggregation [14]. Combination of these functions plays a central role in the cellular stress response to maintain protein homeostasis in eukaryotes [15], [16], [17].

It has been well studied that molecular chaperones and their co-chaperones work together to regulate diverse cell activities including protein folding, transportation and degradation [18]. Among the various co-chaperones, the J-domain (JD) containing proteins (J proteins) are the most popular. There are at least 50 J proteins encoded by the human genome [19]. Homo sapiens J domain protein (HSJ1), also called DNAJB2, is a JD-containing co-chaperone and preferentially expressed in neural tissues [20]. There are two alternatively spliced isoforms identified, HSJ1a and HSJ1b. HSJ1a has been reported to function in regulating the ATPase activity of HSP70 and substrate binding [21]. Besides the conserved J-domain, HSJ1a also harbors two Ub-interacting motifs (UIMs), which make it a special co-chaperone implicated in linking the molecular chaperone and ubiquitination associated degradation [15], [22].

Ataxin-3 (Atx3) is a well known polyQ-containing protein; abnormal expansion of the polyQ tract is responsible for spinocerebellar ataxia type 3 (SCA3) or Machado–Joseph disease [23], [24]. There are literatures reported that overexpression of polyQ-expanded Atx3 may cause heat shock response, and some chaperones and co-chaperones function in orchestrating the protein levels and cellular toxicity of polyQ-expanded Atx3 [9], [25], [26]. For example, HSP40 and HSP70 were reported to localize in the intranuclear aggregates formed by mutant ataxin-3 and that overexpression of HSP40 reduces aggregation of truncated and full-length Atx3 [9]. In this research, we applied exogenous overexpressed Atx3, normal with 22 glutamines (Atx3_22Q_) or polyQ expanded (Atx3_71Q_), to mimic the high protein level of Atx3 in cells and to investigate the function of HSJ1a in cell stress response through potential cooperation between HSP70 chaperone and ubiquitination associated degradation. We found that HSJ1a can dually regulate the proteasomal degradation of the cellular overexpressed Atx3 through maintaining a balance between HSP70 binding and Ub binding. A schematic model for *Yin-Yang* regulating the substrate level by co-chaperones in stressed eukaryotic cells is also proposed and discussed.

## Results

### HSJ1a dually regulates the protein level of Atx3

**Figure 1:**
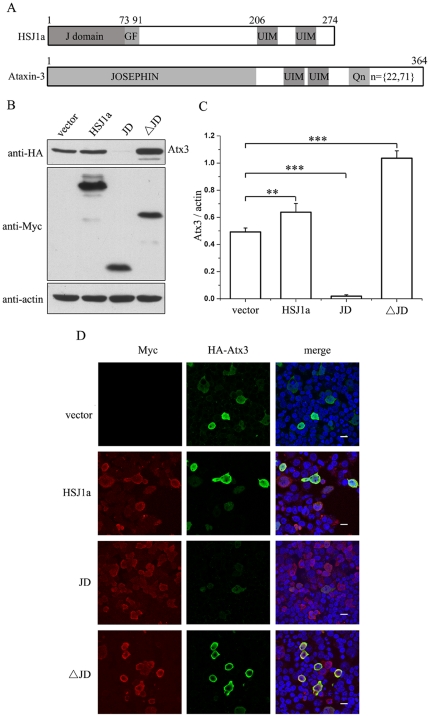
HSJ1a dually regulates the protein level of Atx3 in cells. A, Domain architectures of HSJ1a and Atx3. A polyQ expansion is sometimes occurred in the pathogenic Atx3. B, Effects of HSJ1a and its fragments on the protein levels of normal Atx3. HA-tagged Atx3_22Q_ and Myc-HSJ1a or its fragments (with half DNA amount) were co-transfected into HEK 293T cells. About 48 hrs after transfection, the cell lysates were subjected to immunoblotting with anti-HA, anti-Myc or anti-actin antibody. Atx3 (Atx3_22Q_), normal Atx3 with a polyQ tract of 22 Gln residues; JD, the N-terminal J-domain fragment (residues 1-91); ΔJD, the C-terminal JD-deleted fragment (91-274). C, Quantitative analysis of the Atx3_22Q_ level. The data are from (B) by using *Scion* Image and shown as means ± S.D. (n = 3). ** p<0.01, *** p<0.001. D, Visualization of the cellular protein levels of Atx3 regulated by HSJ1a and its fragments by immuno-fluorescence microscopic imaging. Atx3 is stained with anti-HA antibody (green), HSJ1a and its fragments are stained with anti-Myc antibody (red), and nuclei are stained with Hoechst (blue). Scale bar = 20 µm.

HSJ1a is a co-chaperone that was reported to interact with HSP70 and regulate its chaperoning activities [21]. It is predominantly comprised of an N-terminal HSP70-binding J-domain (JD) and two putative Ub-interacting motifs (UIMs) in the C-terminus (Fig. 1A). To get insights into the possible mechanism underlying that HSJ1a regulates cellular metabolism and affects the protein level of Atx3, we focused the research on the protein level of Atx3 in cellular models by Western blotting and microscopic imaging experiments (Fig. 1). As a result, HSJ1a can slightly increase the protein level of normal Atx3 (Atx3_22Q_) as compared with the mock vector (Fig. 1B & 1C), suggesting that HSJ1a may have regulatory function on the fate of Atx3 via some yet unidentified pathways. To address this issue, we prepared two HSJ1a fragments, JD (residues 1-91) and JD-deletion (ΔJD, 91-274), respectively (Supplemental Fig. S1A). Co-expression with the JD fragment dramatically decreases the protein level of Atx3 to an undetectable extent (Fig. 1B & 1C). On the other hand, co-expression with the ΔJD fragment largely increases the level of Atx3. Confocal microscopic imaging exhibits that the JD fragment of HSJ1a can almost eliminate cellular Atx3, whereas the ΔJD fragment significantly increase the Atx3 amount (Fig. 1D). These results demonstrate that HSJ1a dually regulates the steady state of Atx3 level through both the J-domain that decreases the Atx3 amount and the UIM domain that increases it.

To get information whether the change of Atx3 amount caused by HSJ1a or its fragments in cells is due to the different protein expression efficiency, we examined the transcriptional levels of the constructs by measuring the mRNA amounts (Fig. S1B). Although the mRNA amount of Atx3 exhibits a slight increase in the presence of the JD fragment as compared with others, the difference in mRNA levels cannot account for the remarkable decrease of Atx3 amount caused by the J-domain.

### Chaperone binding is important for the function of the J-domain

**Figure 2:**
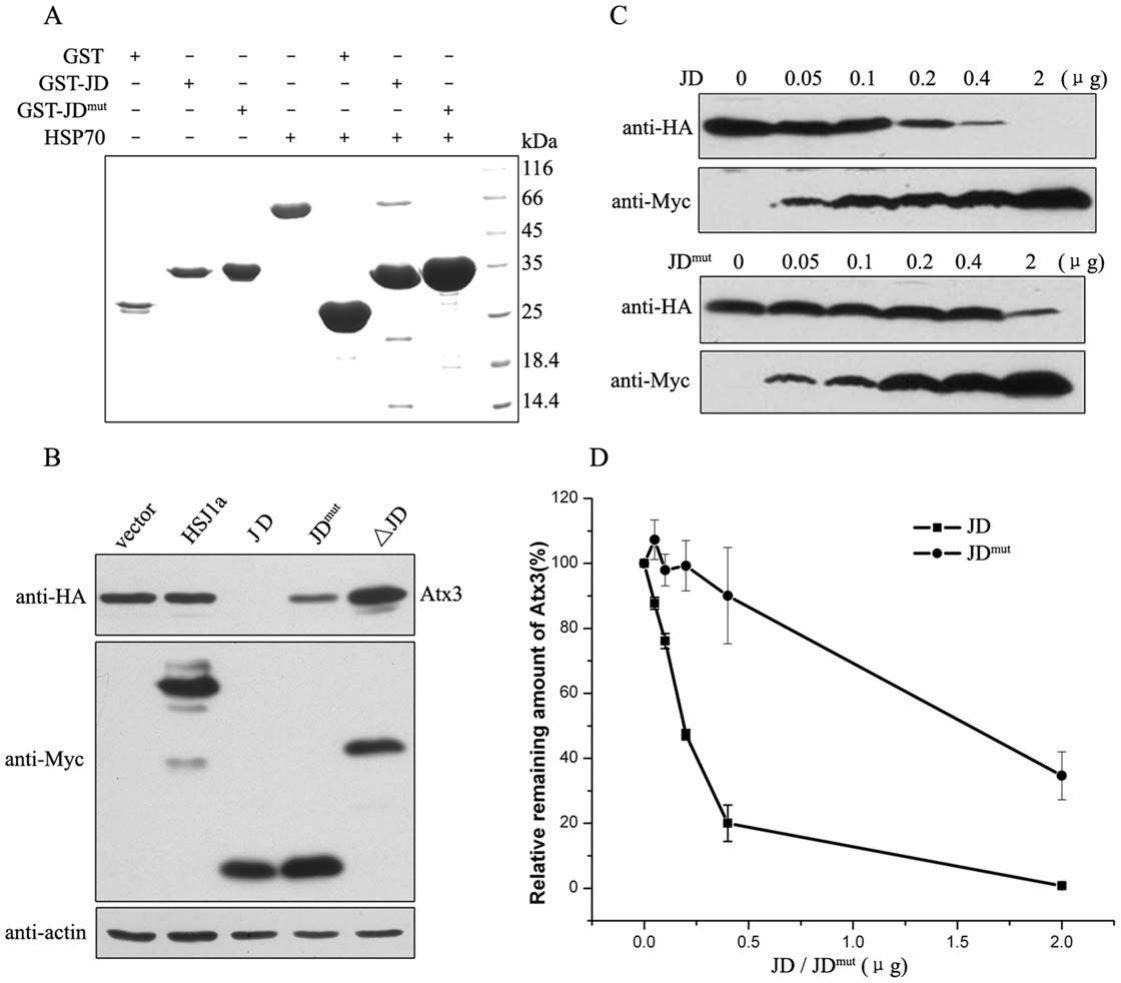
The J-domain of HSJ1a decreases the protein level of Atx3 through its chaperone binding. A, Interaction of HSJ1a with HSP70 by GST pull-down experiment. GST fused J-domain or its mutant (JD^mut^, H31Q/D33N) was applied to pull down HSP70, and GST was as a control.B, Effects of JD and its mutant on the Atx3 levels. Equal amounts of the plasmids encoding HSJ1a or its fragments and Atx3_22Q_ were co-transfected into HEK 293T cells. After transfection (48 hrs), the protein levels were detected by immunoblotting. C, The protein level of Atx3_22Q_ affected by theJ-domain is dose-dependent. The Atx3_22Q_ amounts were assayed by Western blotting under co-expression of different dose of JD or JD^mut^ in HEK 293T cells. D, Quantitative analysis of the Atx3_22Q_ level. The data are from (C) by using *Scion* Image and shown as means ± S.D. (n = 3).

**Figure 3:**
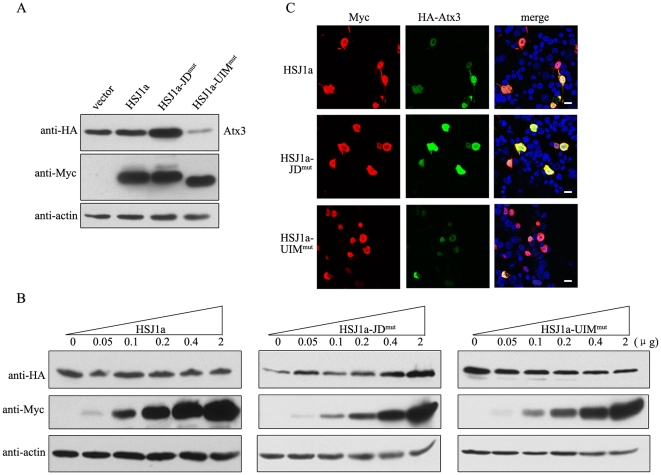
Effects of HSJ1a and its mutants on the Atx3 levels. A, Different effects of HSJ1a and its mutants on the Atx3 level. HA-Atx3_22Q_ and Myc-HSJ1a or its mutants (with half DNA amount) were co-transfected into HEK 293T cells, and then 48 hrs after transfection the cell lysates were subjected to immunoblotting with anti-HA and anti-Myc antibodies. B, Effects of HSJ1a and its mutants on the Atx3 levels by dose-dependent analysis. HA-Atx3_22Q_ and different dose of Myc-HSJ1a or its mutants were co-transfected to HEK 293T cells. The DNA amounts of HSJ1a or its mutants are 0, 0.05, 0.1, 0.2, 0.4 and 2 µg, respectively. C, Immuno-fluorescence imaging of the cellular protein levels of Atx3 regulated by HSJ1a and its mutants. Atx3 is stained with anti-HA antibody (green), HSJ1a and its mutants are stained with anti-Myc antibody (red), and nuclei are stained with Hoechst (blue). Scale bar = 20 µm.

Since HSJ1a dually regulates the protein level of Atx3 by harboring two functional domains, we explored whether this dual regulation is dependent on the binding to chaperone or Ub. The physical interaction between HSJ1 and HSP70 has been studied *in vitro* [21], [27]. Our study indicates that the J-domain of HSJ1a is sufficient to bind with HSP70 (data not shown). The binding site of HSP70 on the J-domain has been well defined to the conserved HPD tripeptide [28], [29]. We replaced the HPD site with QPN on the J-domain of HSJ1a (hereafter referred to JD^mut^, Fig. S1A). GST pull-down experiment shows that this mutation disrupts the interaction between HSJ1a and HSP70 (Fig. 2A). In contrast to the wild-type J-domain, JD^mut^ loses the ability to reduce the protein level of Atx3 (Fig. 2B, lane 4). Moreover, co-expression of JD with Atx3 decreases the amount of Atx3 at a dose-dependent manner (Fig. 2C & 2D). However, JD^mut^ without HPD site largely weakens this reducing effect. This means that interaction between the J-domain and HSP70 chaperone is essential for the HSJ1a functionality. Then, we generated the same HPD-to-QPN mutation on full-length HSJ1a (referred HSJ1a-JD^mut^). In comparison with JD^mut^, this HSJ1a mutant also loses the ability to reduce the Atx3 amount in cells (see Fig. 3A, lane 3), which exhibits similar effect with the ΔJD fragment (Fig. 1B). Taken together, these results suggest that the regulatory function of the J-domain on Atx3 level is dependent on its specific interaction with the HSP70 chaperone.

### Ub binding is important for the function of the UIM domain

Next, we tested whether the increased level of Atx3 by HSJ1a is dependent on the interaction between the UIM domain and Ub chains. The C-terminal UIM domain of HSJ1a contains two UIM motifs (UIM12, residues 206-272) (Fig. 1A). We studied the interaction of UIM12 with isopeptide-linked diUb chains *in vitro* by using biophysical techniques. The data from isothermal titration calorimetry (ITC) shows that UIM12 binds to K48-diUb with a higher affinity than to K63-diUb (Fig. S2A & S2B). NMR titration indicates that the second UIM (UIM2) is more important than the first UIM (UIM1) for binding with K48- or K63-diUb (Fig. S2C, S2D & S2E), which is consistent with the previous report [30]. Then we substituted the two conserved Ser and Glu residues with Ala in each UIM in the context of full-length HSJ1a (referred to HSJ1a-UIM^mut^, Fig. S1A). Co-IP experiment shows that this mutation significantly disrupts the interaction with polyUb chains in cells (Fig. S2F). The UIM mutation indeed abolishes the increasing effect of HSJ1a on the protein level of Atx3 (Fig. 3A, lane 4), exhibiting similar effect with the JD fragment that significantly decreases the level of Atx3 (Fig. 1B). These effects on the Atx3 levels are also confirmed by the experiments under an HSJ1a dose-dependent manner (Fig. 3B). Immuno-fluorescence imaging also directly shows that HSJ1a-JD^mut^ significantly increases the protein level of Atx3, whereas HSJ1a-UIM^mut^ considerably decreases it (Fig. 3C). These results clearly indicate that regulation of Atx3 by HSJ1a is somehow related to the binding of its C-terminal UIM domain to Ub chains.

### HSJ1a regulates degradation of Atx3 through a proteasome pathway

**Figure 4:**
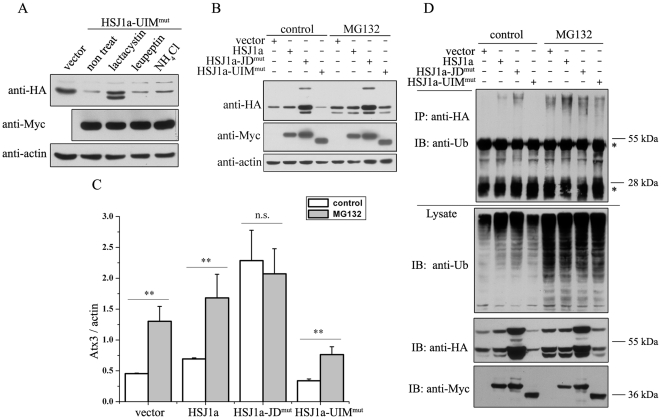
HSJ1a regulates the proteasomal degradation of Atx3. A, Effects of proteasome or lysosome inhibitors on the HSJ1a-modulated protein level of Atx3. Equal amounts of HA-Atx3 and Myc-HSJ1a-UIM^mut^ or mock vector were co-transfected into HEK 293T cells. After transfection (36 hrs), the cells were treated with 10 µM lactacystine or leupeptin (proteasome inhibitor) or 2 mM NH_4_Cl (lysosome inhibitor) for 10 hrs. Then the cell lysates were subjected to immunoblotting with anti-HA, anti-Myc or anti-actin antibody. B, Effect of proteasome inhibitor MG132 on the HSJ1a-modulated protein level of Atx3. Equal amounts of HA-Atx3 and Myc-HSJ1a, Myc-HSJ1a-JD^mut^, Myc-HSJ1a-UIM^mut^ or mock vector were co-transfected into HEK 293T cells. After 36 hrs, the cells were treated with 10 µM MG132 for 10 hrs. C, Quantitative analysis of the Atx3_22Q_ level. The data are from (B) by using *Scion* Image and shown as means ± S.D. (n = 3). ** p<0.01. D, Ubiquitination of Atx3 affected by HSJ1a and its mutants. The cell lysates as shown in (B) were subjected to immunoprecipitation with anti-HA antibody and the resulting precipitates were subjected to immunoblotting with anti-Ub antibody (upper panel). The total ubiquitination of the lysates is shown as a control (middle panel). The asterisks denote the bands from the heavy and light chains of IgG.

Since regulation of Atx3 by HSJ1a is not possibly related to the transcriptional way (Fig. S1B), the fluctuation in the Atx3 amount is somehow associated with the degradation that may be modulated by HSJ1a. Previous studies indicate that Atx3 is degraded by the Ub-proteasome pathway [31]. We examined whether the J-domain of HSJ1a promotes the degradation of Atx3 through a proteasome or lysosome pathway. We treated the HEK 293T cells that overexpress Atx3 and HSJ1a-UIM^mut^ with a proteasome or lysosome inhibitor, and then examined the Atx3 levels of the cell lysates. In the presence of the proteasome inhibitor lactacystine, the Atx3 level is recovered to that as the control vector only (Fig. 4A). However, the lysosome inhibitor, ammonium chloride (NH_4_Cl) has only a slight influence on the Atx3 level, similar to non-treatment of the cells. In the cells transfected with Atx3 and HSJ1a or HSJ1a-UIM^mut^, inhibition of proteasome activity by MG132 results in a remarkable accumulation of Atx3 as in the case of mock vector (Fig. 4B & 4C). However, proteasome inhibition has no considerable effect on the amount of Atx3 when the cells were transfected with Atx3 and HSJ1a-JD^mut^ (Fig. 4C), because HSJ1a-JD^mut^ has exerted the effect on accumulating Atx3 protein to a saturation before the proteasome activity being inhibited. Thus we propose that the degradation of Atx3 modulated by HSJ1a may undergo through a proteasome pathway.

As known, ubiquitination of a protein substrate is required for its proteasomal degradation [32], [33], thus the degradation of Atx3 modulated by HSJ1a may also be associated with ubiquitination of Atx3. To test this hypothesis, we co-transfected Atx3 and HSJ1a or its mutants into HEK 293T cells, treated the cells with proteasome inhibitor MG132, and then detected the ubiquitination level of Atx3 by co-immunoprecipitation. As a result, both HSJ1a and HSJ1a-JD^mut^ can promote accumulation of the ubiquitinated Atx3 in cells (Fig. 4D), as compared with the mock vector. However, the HSJ1a-UIM^mut^ loses the ability to accumulate the ubiquitinated Atx3, which can only be detected in the presence of MG132. Taken together, these results demonstrate that HSJ1a down-regulates the degradation of Atx3 through accumulating the ubiquitinated Atx3 involved in the Ub-proteasome pathway.

Since Atx3 itself also harbors two UIM motifs near the polyQ region that may also recruit ubiquitinated substrates (Fig. 1A), there is still a possibility that the ubiquitinated proteins we have observed in the gels are not Atx3 itself but other substrates. To exclude this possibility, we prepared some mutations in the UIM region of Atx3 (S236A/S256A, Atx3-UIM^mut^) to disrupt its binding with Ub chains [34], [35]. The results show that HSJ1a exerts the similar effect on the UIM mutant of Atx3 (Fig. S3). Therefore, we conclude that the UIM domain of HSJ1a plays an important role in protecting the ubiquitinated Atx3 from proteasomal degradation.

### Atx3 is a substrate of HSP70-mediated proteasomal degradation

**Figure 5:**
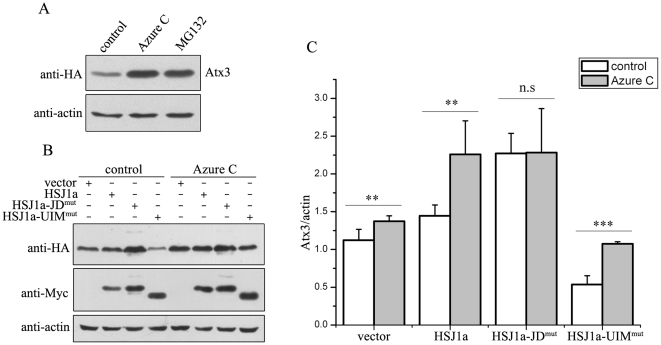
HSP70 is directly involved in the HSJ1a-modulated proteasomal degradation of Atx3. A, Effect of inhibiting the ATPase activity of HSP70 on the Atx3_22Q_ level. HA-Atx3_22Q_ was transfected into HEK 293T cells, and then 24 hrs after transfection the cells were treated with 50 µM Azure C or 20 µM MG132 for 10 hrs. The cell lysates were subjected to immunoblotting with anti-HA antibody. B, Effects of HSP70 inhibition on the HSJ1a-modulated Atx3 levels. HA-Atx3_22Q_ and Myc-tagged HSJ1a, HSJ1a-JD^mut^, HSJ1a-UIM^mut^ or mock vector were co-transfected into HEK 293T cells, and 24 hrs later, the cells were treated with 50 µM Azure C or MG132 for 10 hrs. C, Quantitative analysis of the Atx3_22Q_ level. The data are from (B) by using *Scion* Image and shown as means ± S.D. (n = 3). ** p<0.01; *** p<0.001.

As the J-domain binds with HSP70 chaperone and promotes degradation of Atx3 (Fig. 2), it is most likely that HSJ1a regulates the Atx3 level though an HSP70-mediated degradation pathway. It has been established that HSP70 and its co-chaperones regulate the proteasomal degradation of some short-lived or aberrant proteins, such as polyQ-containing proteins [9], [36], [37]. To investigate whether HSP70 is a direct mediator for degradation of Atx3 modulated by HSJ1a, we treated the cells with ATPase inhibitors of HSP70 [38]. As shown, Azure C treatment does lead to a significant increase of Atx3 (Fig. 5A). Methylene blue (MB), another HSP70 inhibitor proved to inhibit the ATPase activity and to regulate degradation of polyQ-containing proteins [39], has the similar effect on the Atx3 amount (data not shown). Interestingly, Azure C treatment exhibits similar effect with MG132 treatment, both of which lead to accumulation of the Atx3 protein (Fig. 5A & 5B). Similar to inhibition of proteasome, inhibition of the HSP70 activity results in a significant accumulation of Atx3 when co-transfected with mock vector, HSJ1a or HSJ1a-UIM^mut^, but it has no such effect on the cells co-transfected with HSJ1a-JD^mut^, a mutant with co-chaperone deficiency (Fig. 5B & 5C). These results suggest that HSP70 is directly involved in the degradation of Atx3 modulated by HSJ1a.

### CHIP is also involved in the HSJ1a-modulated degradation of Atx3

**Figure 6:**
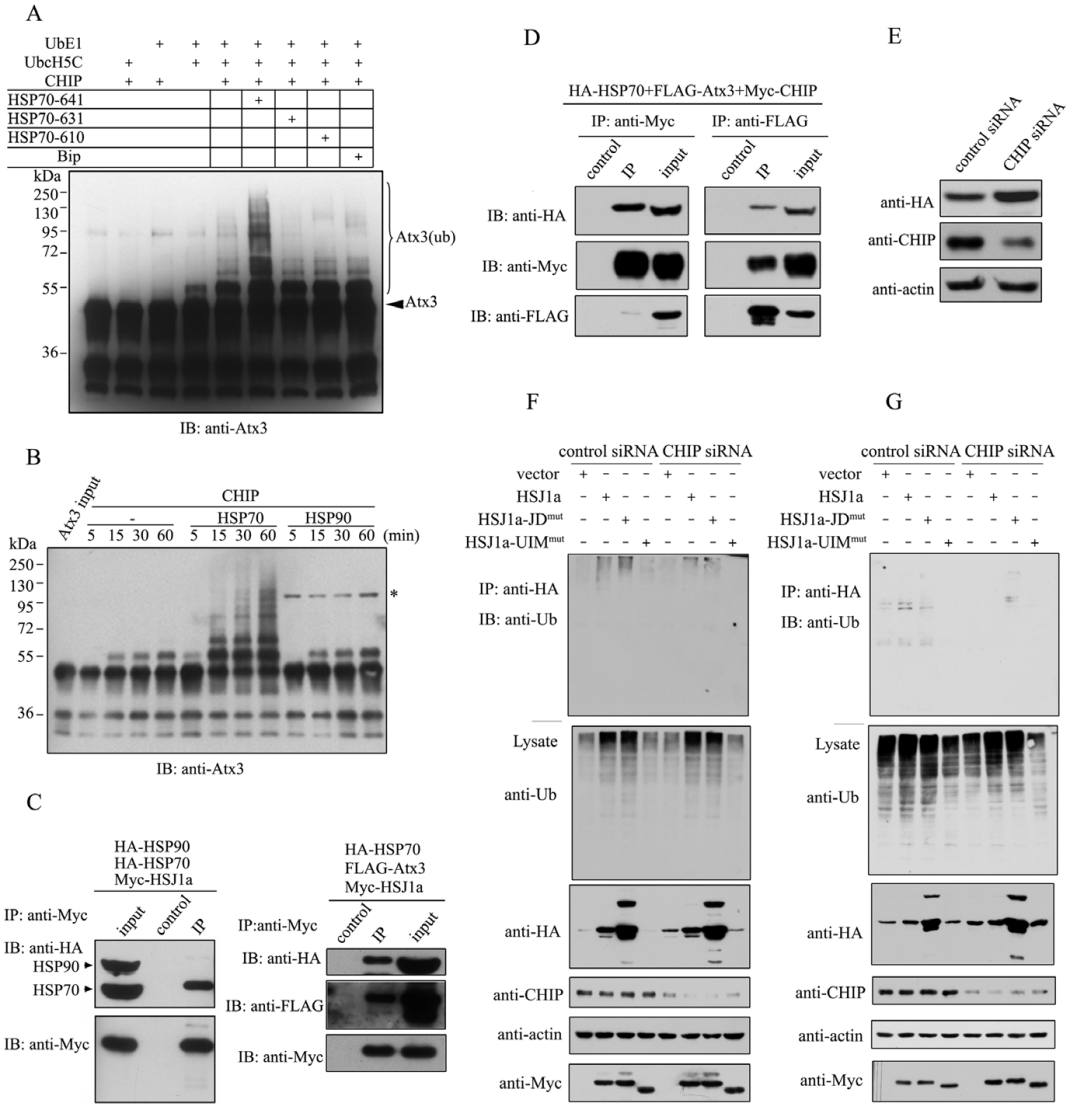
HSP70 and CHIP mediate the proteasomal degradation of Atx3. A, *In vitro* ubiquitination of Atx3 promoted by HSP70 and CHIP. Atx3 (arrow indicated) and its ubiquitinated forms (bracket indicated) were detected by immunoblotting with an anti-Atx3 antibody. B, *In vitro* ubiquitination of Atx3 mediated by HSP70 and HSP90. The reaction products were detected as in (A). The GB1-tagged HSP90 was used in this study for the sake of protein stability. The asterisk denotes GB1-HSP90, in which GB1 can be detected by the antibody. C, Co-IP experiments for interactions of HSJ1a with HSP70 or HSP90 and Atx3. HEK 293T cells were co-transfected with HSJ1a-Myc, HA-HSP70 and HA-HSP90 or FLAG-Atx3, the cell lysates were subjected to co-immunoprecipitation. IP, anti-Myc antibody; IB, anti-HA, anti-Myc and anti- FLAG antibodies. D, Co-IP experiments for the ternary complex among interactions of CHIP, HSP70 and Atx3. HEK 293T cells were co-transfected with HSJ1a-Myc or Myc-CHIP, HA-HSP70 and FLAG-Atx3, and then the cell lysates were subjected to co-IP experiment. IP, anti-Myc or anti-FLAG antibody; IB, anti-HA, anti-FLAG and anti-Myc antibodies. E, Knockdown of CHIP increases the level of overexpressed Atx3. HEK 293T cells were transfected with scrambled or CHIP siRNA and HA-Atx3. The protein levels of Atx3 and endogenous CHIP were measured by immunoblotting with anti-HA and anti-CHIP antibodies. F & G, knockdown of CHIP lowers down the amount of ubiquitinated Atx3 (F) or Atx3-UIM^mut^ (G) maintained by HSJ1a or HSJ1a-JD^mut^. HEK 293T cells were transfected with scrambled or CHIP siRNA and indicated expression vectors. The cell lysates were subjected to immunoprecipitation with anti-HA antibody and the resulting precipitates were subjected to immunoblotting with anti-Ub antibody (upper panel). The lysates were also subjected to immunoblotting with anti-HA, anti-CHIP, anti-actin and anti-Myc antibodies.

CHIP is an E3 or E4 ligase for ubiquitination; its TPR domain can interact with the C-terminal EEVD motif of HSP70 [40], [41], [42]. Many studies on the function of CHIP have linked HSP70 to the Ub-proteasome system [26], [43], [44], [45]. It was previously reported that overexpression of CHIP increases the ubiquitination and degradation rates of polyQ-expanded Atx3, which is also enhanced by HSP70 [26]. This indicates that Atx3 is a potential substrate of HSP70. Indeed, our data show that overexpression of HSP70 or CHIP leads to the up-regulation of the ubiquitination level and down-regulation of the Atx3 level (Fig. S4). Without HSP70, CHIP can ubiquitinate Atx3 *in vitro*, but with a rather low efficiency. Interestingly, HSP70 can dramatically enhance the ubiquitination efficiency of Atx3 (Fig. 6A). Deletion of the CHIP-binding motif in the C-terminus of HSP70 (HSP70-631 or HSP70-610, residues 1-631 or 1-610) results in loss of the ability of CHIP as an E3 ligase to promote ubiquitination of Atx3. Besides HSP70, HSP90 is another chaperone also harboring an EEVD motif for CHIP binding. However, HSP90 has no such effect on promoting ubiquitination (Fig. 6B). Actually, HSJ1a is prior to binding with HSP70 when both chaperones are present in cells (Fig. 6C), even though HSJ1a can interact with HSP70 and HSP90, respectively (Fig. 2A, Fig. S5) [27]. These results suggest that down-regulation of the Atx3 level by the J-domain of HSJ1a is mediated by HSP70 rather than HSP90. Next, we tested whether HSJ1a, CHIP, HSP70 and Atx3 can form a complex in cells. We overexpressed FLAG-Atx3, HA-HSP70 and Myc-HSJ1a or Myc-CHIP in HEK 293T cells for co-IP experiments. The data show that HSJ1a can precipitate both HSP70 and Atx3 (Fig. 6C), while CHIP can also precipitate HSP70 but a trace amount of Atx3 (Fig. 6D). This may be attributable to the quantitative difference and transient interaction between the substrates and E3 ligase. A reverse co-IP experiment shows that a considerable amount of CHIP can be precipitated by Atx3 (Fig. 6D). These results provide a possibility that HSP70 and its co-chaperones, HSJ1a and CHIP, work together to regulate the degradation of Atx3.

Although our data show that CHIP may associate with Atx3 to ubiquitinate Atx3 *in vitro*, we still wonder whether CHIP is directly involved in the degradation of Atx3. We thus knocked down CHIP expression by using siRNA in HEK293T cells. As a result, silencing of CHIP significantly increases the amount of Atx3 (Fig. 6E), suggesting that CHIP may down-regulate the Atx3 level. Next we examined whether CHIP regulates Atx3 through HSJ1a. When co-transfected with HSJ1a or its mutants, the amount of Atx3 increases in the context of CHIP silencing (Fig. 6F, 3^rd^ panel). A decrease of the ubiquitinated conjugates precipitated by Atx3 is also observed as compared with the siRNA control (1^st^ panel). To further demonstrate this observation, we used an Atx3 mutant (Atx3-UIM^mut^) to repeat this experiment. As mentioned above (Fig. S3), Atx3-UIM^mut^ loses the potential to bind with polyUb chains, so the ubiquitinated conjugates precipitated by this mutant are mostly the ubiquitinated form of Atx3. Indeed, the amount of the ubiquitinated conjugates, which was maintained by HSJ1a, was significantly reduced when CHIP silencing (Fig. 6G). Collectively, these results demonstrate that CHIP is directly involved in the degradation of Atx3 regulated by HSJ1a.

### HSJ1a can also dually regulate polyQ-expanded Atx3 through HSP70-assisted proteasomal degradation pathway

**Figure 7:**
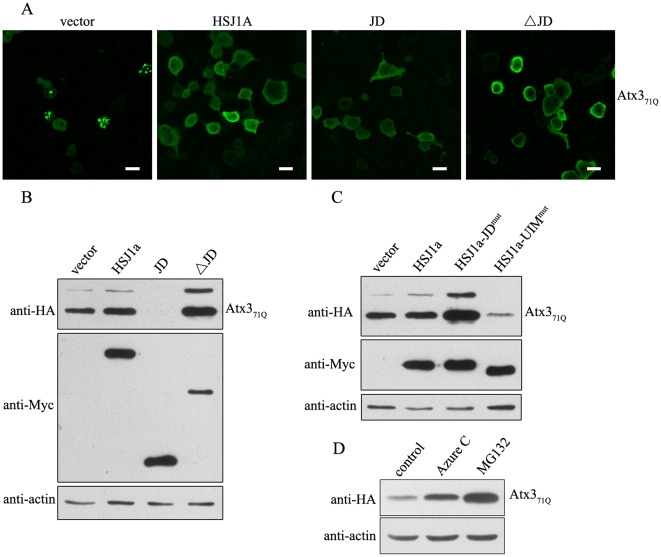
HSJ1a can also dually regulate the protein level of the polyQ-expanded Atx3 (Atx3_71Q_) in cells. A, Immuno-fluorescence imaging of the cellular inclusions formed by Atx3_71Q_ regulated by HSJ1a and its fragments. B, Effects of HSJ1a and its fragments on the protein levels of Atx3_71Q_. HA-tagged Atx3_71Q_ and Myc-HSJ1a or its fragments (with half DNA amount) were co-transfected into HEK 293T cells. About 48 hrs after transfection, the cell lysates were subjected to immunoblotting with anti-HA, anti-Myc or anti-actin antibody. Atx3_71Q_, polyQ-expanded Atx3 with 71 Gln residues. C, Different effects of HSJ1a and its mutants on the protein level of Atx3_71Q_. HA-Atx3_71Q_ and Myc-HSJ1a or its mutants (with half DNA amount) were co-transfected into HEK 293T cells, and then 48 hrs after transfection the cell lysates were subjected to immunoblotting with anti-HA and anti-Myc antibodies. D, Effect of inhibiting the ATPase activity of HSP70 on the Atx3_71Q_ level. HA-Atx3_71Q_ was transfected into HEK 293T cells, and then 24 hrs after transfection the cells were treated with 50 µM Azure C or 20 µM MG132 for 10 hrs. The cell lysates were subjected to immunoblotting with anti-HA antibody.

It is generally accepted that polyQ expansion of proteins is associated with the pathogenesis of some neurodegenerative diseases [46]. There is a report that HSJ1a can reduce polyQ-mediated inclusion body formation in a model of spinal and bulbar muscular atrophy (SBMA) [47]. Similarly, we also observed in HEK 293T cells that endogenous HSP70 and HSJ1 are recruited into the nuclear inclusion bodies formed by polyQ-expanded Atx3 (Atx3_71Q_) (Fig. S6). To understand whether HSJ1a regulates the metabolism of the pathogenic form of Atx3, we performed the experiments on the polyQ-expanded Atx3_71Q_. Overexpression of HA-tagged Atx3_71Q_ causes inclusion body formation in cells (Fig. 7A), which can be alleviated by overexpression of HSJ1a, the JD or ΔJD fragment, although both HSJ1a and its ΔJD fragment increase the Atx3_71Q_ levels (Fig. 7B). As in the case of Atx3_22Q_ (Fig. 1, 2 & 3), the cellular protein levels of Atx3_71Q_ are also modulated by HSJ1a and its fragments (Fig. 7B & 7C), in which deletion and mutation in the J-domain or UIM domain of HSJ1a give rise to similar effect on the protein levels of Atx3_71Q_ in cells (Fig. S7). Azure C or MG132 that inhibit HSP70 function or proteasome activity can also significantly increase the protein level of Atx3_71Q_ (Fig. 7D), as in the case of Atx3_22Q_ (Fig. 4 & 5). All these experimental data demonstrate that, similar with that of normal Atx3, the protein level of pathogenic Atx3 is also regulated by HSJ1a through an HSP70-assisted proteasomal degradation pathway. This study may provide a possibility therapeutically for eliminating the cellular inclusion bodies formed by polyQ-expanded Atx3 though interfering with the functionality of the two domains in the HSJ1a co-chaperone.

## Discussion

### Overexpression of Atx3 and cell stress response

In eukaryotic cells, the amount of a protein must be maintained to a relatively stable level by diverse regulatory processes. Loss of this regulation may cause protein accumulation and even abnormal aggregation or inclusion formation, and consequently lead to stress response [2], [48]. It was previously reported that overexpression of abnormal Atx3 can evoke a stress response in cells as manifested by marked induction of HSP70 [9]. The overexpressed Atx3 may cause a proteotoxic stress in cells and it will be eliminated (or refolded) by HSP70 chaperone machinery. Thus, it is very important for a cell to respond the overexpression stress. Molecular chaperones such as HSP70 may act as a sensor of the abnormal proteins by recognize the misfolded or aggregated proteins [2]. The data from our study and other literatures implicate that the overexpressed Atx3 is a potential substrate of HSP70 and thus orchestrated by HSJ1a [26], [49]. However, HSJ1a cannot regulate the endogenous Atx3 (Fig. S8), possibly because Atx3 with normal amount is correctly folded that cannot be censored by HSP70. Thus, we propose that HSP70 is a key molecular factor to censor the overexpressed Atx3 and trigger the cell stress response.

### HSJ1a binds HSP70 and promotes proteasomal degradation of Atx3

HSJ1a is a J-domain containing co-chaperone of HSP70 that binds with HSP70 and stimulates its ATPase activity for modulating its functionality [21]. Molecular chaperones and their co-chaperones, such as HSP70, HSP40 and CHIP, may function in modulating aggregation, cytotoxicity, and disease onset in polyQ diseases [9], [25], [26], [50], [51], [52]. Recently, HSP70 and CHIP have been proposed to regulate the proteasomal degradation of polyQ-expanded proteins including Atx3 [26], [49]. Our studies both *in vitro* and in cell model demonstrate that the N-terminal J-domain of HSJ1a binds HSP70 and enhances proteasomal degradation of Atx3 (Fig. 3 & 7). HSP70 dramatically promotes the ubiquitination efficiency of overexpressed Atx3 in the presence of CHIP (Fig. 6), while inhibition of the ATPase activity of HSP70 as well as proteasome significantly accumulates the ubiquitinated Atx3 (Fig. 4 & 7). Therefore, the overexpressed Atx3, whether it is normal or polyQ expanded, is an HSP70 client and subsequently the substrate of HSP70-assisted proteasomal degradation. As a co-chaperone of HSP70, HSJ1a functions as a modulator to regulate this process.

### HSJ1a binds ubiquitinated Atx3 and retards its degradation

HSJ1a also harbors two UIM motifs that may function in binding with Ub chains or ubiquitinated substrates. Our observation indicates that the UIM domain of HSJ1a can accumulate the ubiquitinated Atx3 and impede it from proteasomal degradation (Fig. 4, 5 & 7). This is generally consistent with the previous observation that HSJ1a can accumulate ubiquitinated proteins [47]. As known, the J-domain of HSJ1a rather than the UIM domain exerts a function down-regulating the protein level of Atx3. The UIM domain of HSJ1a actually finely decelerates the degradation process by accumulating the ubiquitinated Atx3.

It was reported that HSJ1a can stimulate CHIP-mediated ubiquitination of Raf-1, and the UIM domain is not responsible for this stimulation [30]. Our *in vitro* study indicates that HSJ1a can inhibit the CHIP-mediated ubiquitination of Atx3 depending on the UIM domain, whereas the J-domain cannot promote the process (Fig. S9). It is possible that the J-domain of HSJ1a enhances the degradation of Atx3 through assisting release of the ubiquitinated Atx3 from the HSP70-CHIP complex, instead of promoting its ubiquitination. The UIM domain may prevent the degradation of Atx3 through binding with the ubiquitinated Atx3 and inhibiting its further ubiquitination in cells.

### HSJ1a is a dual regulator of the cellular Atx3 level

One of the important issues involved in HSP70-assisted proteasomal degradation is that how a chaperone cooperates with UPS. Actually, it is the co-chaperones that act as bridges to link these two systems. Among the diverse co-chaperones of HSP70 yet identified, several proteins harbor both chaperone and Ub related domains [14]. Besides HSJ1 (JD-UIM) in this study, these proteins include BAG-1 (BAG-UbL) [53], BAG-6/BAT3 (UbL-BAG) [54], Sacsin (JD-UbL) [55], and the well-characterized CHIP (TPR-Ubox) [56]. These co-chaperones have great potential to play important roles in the protein quality control processes through linking the chaperone system and UPS. In this study, we demonstrate that co-chaperone HSJ1a can dually regulate degradation of Atx3, and even eliminate the inclusion bodies formed by polyQ expanded Atx3 (Fig. 7A) and another polyQ disease-related protein Atx7 (data not shown) in cells. The J-domain and the UIM domain play distinct roles in this particular process. Interestingly, the increasing level of polyQ-expanded Atx3 caused by the ΔJD fragment does not result in an increase of the inclusions (Fig. 7A).

### HSJ1a regulates the Atx3 level in a *Yin-Yang* manner

**Figure 8:**
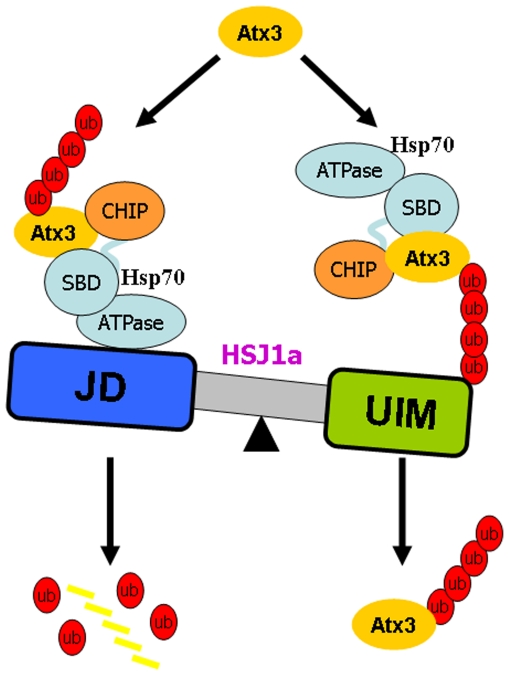
Schematic representation of the proteasomal degradation of Atx3 showing different roles of the two domains of HSJ1a. This model proposes that HSJ1a acts like a balance orchestrating the cellular level of Atx3. The J-domain promotes the proteasomal degradation of Atx3 through HSP70 binding, whereas the UIM domain retards the proteasomal degradation of Atx3 through ubiquitin binding. HSP70 acts as an adaptor to recruit co-chaperones and the substrates.

Our findings demonstrate that the N-terminal J-domain of HSJ1a promotes proteasomal degradation of Atx3, whereas the C-terminal UIM domain alleviates the degradation. In this regard, HSJ1a may exert a role deciding whether a chaperone client to be degraded in proteasome or to be stayed in its ubiquitinated form [14]. Thus, we propose that HSJ1a dually regulates the protein level of Atx3 in stressed cells in a *Yin-Yang* manner, no matter it is normal or polyQ expanded (Fig. 8). In this model, the chaperone HSP70 recruits the E3 ligase CHIP to ubiquitinate Atx3, while the co-chaperone HSJ1a enhances the function of the HSP70 complex to eliminate the overexpressed substrate. The J-domain of HSJ1a binds with HSP70 to promote the chaperone-assisted proteasomal degradation of the substrate, as in the case of HSP40 that associates with HSP70 to reduce aggregation of Atx3 [9]. On the other hand, HSJ1a can also impede the proteasomal degradation of the ubiquitinated Atx3 to maintain the protein level when needed. The UIM domain of HSJ1a may bind with and stabilize the ubiquitinated Atx3, so as to protect it from degradation. These two distinct processes make HSJ1a a dual regulator to maintain the homeostasis of Atx3 molecules. Impairment of the protein homeostasis may cause a pathogenic phenotype, for example formation of inclusion bodies [2]. Thus, finely modulating the balance of the functions of two HSJ1a domains could be considered as a promising therapeutic strategy for neurodegenerative diseases [1].

## Materials and Methods

### Construction of the expression plasmids

The coding sequence for HSJ1a was referred to GenBank (AAA09034.1). The cDNAs encoding for HSJ1a (residues 1-274), JD (1-91), ΔJD (91-274), and UIM12 (206-272) were amplified via PCR. The PCR products were cloned into pGEX-4T-3 or pGBTNH [57] vector for bacterial expression or pcDNA3.1-Myc/His vector for eukaryotic expression using *Bam*H I/*Xho* I cloning sites. HSJ1a-JD^mut^ (H31Q/D33N) and HSJ1a-UIM^mut^ (S219A/E222A/S262A/E265A) were prepared using site-directed mutagenesis via PCR technique. The genes encoding for Atx3 (22Q or 71Q) and its mutant (S236A/S256A) [35] were cloned into pcDNA3-HA vector using *Bam*H I/*Xho* I cloning sites to get HA-tagged proteins or cloned into pEGFPN1 vector using *Xho* I/*Bam*H I cloning sites to get GFP-tagged proteins. HSP70 was cloned into pET22b^+^ expression vector to get bacterially expressed proteins and then inserted into pcDNA3-HA to get HA-HSP70. CHIP was cloned to pET22b^+^ vector for bacterial expression or pcDNA3.1-Myc/His for eukaryotic expression. All constructs were confirmed by DNA sequencing.

### Protein expression and purification

The genes encoding His-tagged or GST fusion proteins were expressed in *E. coli* BL21 (DE3) cells. The His-tagged proteins were purified through a Ni^+^-NTA column (Qiagen) according to manufacturer's instructions. GST and the GST fusion proteins were purified using the glutathione Sepharose 4B column (Amersham Biosciences). The ^15^N- and ^15^N/^13^C-labeled GB1-HSJ1a-UIM12 proteins were prepared by using the M9 minimal medium containing ^15^N-NH_4_Cl and/or ^13^C_6_-D-glucose, and were purified by Ni^+^-NTA affinity column. All the proteins were further purified by a Superdex-75 column (GE Healthcare).

### GST pull-down experiment

The purified GST or GST-JD protein was incubated with glutathione Sepharose 4B beads in a PBS buffer (10 mM Na_2_HPO4, 140 mM NaCl, 2.7 mM KCl, 1.8 mM KH_2_PO4, pH 7.3) at 4°C for 1 hr. The His-tagged proteins were incubated with the immobilized GST or GST-fusion proteins at 4°C. After incubating for 2 hrs, the beads were collected by centrifugation and washed 4 times with the same buffer. The samples were then subjected to SDS-PAGE, followed by Coomassie blue staining.

### Cell culture and transfection

HEK 293T cells (American Type Culture Collection, Manassas, VA, USA) were cultured in Dulbecco's modified Eagle's medium (DMEM)/F-12 (Invitrogen) supplemented with 10% fetal bovine serum (Hyclone), penicillin, and streptomycin and grown at 37°C under a humidified atmosphere containing 5% CO_2_ [58]. The cells were transfected with the expression vectors by using FuGENE®HD Transfection Reagent (Roche) following the manufacturer's instructions. MG132 and lactacystine were purchased from Calbiochem. Leupeptin, Azure C and Methylene blue were purchased from Sigma. For inhibition experiment, about 24 hrs after transfection, the cells were treated with different inhibitors for 10∼16 hrs before lysis. The siRNA target sequence for CHIP is 5′-GUGGCAAGAUCAGCUUUGA-3′, and the siRNA control sequence is 5′-UUCUCCGAACGUGUCACGU-3′. The cells were transfected with the duplex siRNA using lipofectamine 2000 (Invitrogen) following the manufacture's instructions.

### Western blotting and antibodies

The cells were harvested 48 hrs after transfection and lysed in RIPA buffer (50 mM Tris–HCl, pH 7.5, 150 mM NaCl, 1 mM EDTA, 1 mM PMSF, cocktail protease inhibitor (Roche), 1% NP-40), and then the lysates were subjected to SDS-PAGE with 10%, 12% or 15% acrylamide gels and transferred onto PVDF membranes (PerkinElmer). The indicated proteins were probed with the following primary antibodies: mouse monoclonal antibodies against HA (Sigma), FLAG (Sigma), Myc (Cell Signaling), His (Sigma), Ub (Santa Cruz) and Atx3 (Chemicon); goat anti-actin antibody (Santa Cruz), rabbit polyclonal anti-Myc antibody (Cell Signaling), rabbit polyclonal anti-HSP70 antibody (Proteintech), rabbit polyclonal anti-CHIP (Sigma), mouse monoclonal antibody against inducible HSP70 (Abcam). The goat anti-mouse IgG-HRP antibody, goat anti-rabbit IgG-HRP and rabbit anti-goat IgG-HRP secondary antibodies (Jackson Immuno-Research) were also used. The proteins were visualized using an ECL detection kit (Amersham Pharmacia Biotech).

### Immunoprecipitation, Immunocytochemistry, and confocal microscopy

The HEK 293T cells were lysed in RIPA buffer for 30 min on ice and then the lysates were incubated with an antibody for 2 hrs at 4°C. After incubation, the protein A/G beads (GE Healthcare) were added for another 4 hrs at 4°C. The beads were washed with lysis buffer for four times, and the precipitated proteins were subjected to immunoblotting analysis.

The HEK 293T cells grown and transfected on cover slides were washed with PBS buffer and fixed with 4% paraformaldehyde for 10 min and permeabilized with 0.1% Triton X-100 for 1 min, and then blocked with 5% BSA/1% FBS for 1 hr at room temperature. The fixed cells were incubated for 1 hr at room temperature with a combination of a monoclonal antibody against HA (Sigma) and a polyclonal antibody against Myc (Cell Signaling). After washing with PBS, the cells were labeled with an FITC-conjugated anti-mouse antibody and a Cyanine 3 conjugated anti-rabbit secondary antibody (Jackson Immuno-Research) [58]. The nuclei were stained with Hoechst (Sigma). The cells were visualized with a Leica TCS SP2 confocal microscope (Leica Microsystems).

### *In vitro* ubiquitination assay

The Atx3-His protein and 0.1 µM mouse UbE1 (previously prepared in this lab), 2.4 µM UbcH5C, 1 µM CHIP, 5 µM Ub, 2 mM ATP with or without 1 µM HSP70 (or HSP90) were mixed in a reaction buffer (50 mM Tris-HCl, 5 mM MgCl_2_, pH 7.6) and incubated for 1 hr at 37°C. The reactions were stopped by adding the sample buffer for SDS-PAGE and then detected by immunoblotting analysis.

## Supporting Information

Figure S1 Effects of HSJ1a and its fragments on the mRNA levels of Atx3. (A) Sequences and domain architecture of HSJ1a and its fragments and mutants. (B) Quantitative real-time PCR analysis of Atx3 mRNA levels in HEK 293T cells transiently transfected with the indicated plasmids as shown. Shown are means ± S.D. (n = 3). JD, J-domain fragment; ΔJD, J-domain deleted fragment.
(PDF)

Figure S2 Interactions between the UIM12 domain of HSJ1a and different diUb chains. (A) ITC experiment for the interaction of GB1-UIM12 with K48-diUb. The concentration of diUb is 100 µM and that of the GB1-UIM12 stock is 2 mM. The data are fitted with the one-site binding model. The GB1 fusion was used for purification and quantification. (B) As (A), with K63-diUb. (C) NMR titration of GB1-UIM12 with K48-diUb. Shown is the overlay of the ^1^H-^15^N HSQC spectra of ^15^N-labeled GB1-UIM12 before (green) and after (red) addition of 1 equiv. of K48-diUb. (D) As (C), with K63-diUb. Shown is the overlay of the ^1^H-^15^N HSQC spectra of ^15^N-labeled GB1-UIM12 before (blue) and after (red) addition of 1 equiv. of K63-diUb. (E) Plot of the relative peak heights against the residue number of GB1-UIM12 titrated with 1 equiv. of K48-diUb. All the peak heights were normalized except for prolines and unassigned residues. The diagram shows the peak heights for the residues from GB1 (yellow), UIM1 (blue), the linker (purple) and UIM2 (blue). (F) Co-IP experiment for interaction between HSJ1a or its UIM mutant and endogenous Ub chains. HEK 293T cells were transiently transfected with a vector expressing His-HSJ1a or His-HSJ1a-UIM^mut^. After 48 hrs, the cells were subjected to immunoprecipitation (IP) with anti-His and the resulting precipitates were subjected to immunoblotting (IB) with either anti-Ub or anti-His. The asterisks denote the bands from the heavy and light chains of IgG.
(PDF)

Figure S3 HSJ1a exerts the similar effect on the UIM mutant form of Atx3. (A) Different effects of HSJ1a and its mutants on the protein levels of Atx3-UIM^mut^. HA-Atx3-UIM^mut^ and Myc-HSJ1a or its mutants were co-transfected into HEK 293T cells. About 48 hrs after transfection, the cell lysates were subjected to immunoblotting with anti-HA and anti-Myc antibodies. (B) Ubiquitination of Atx3-UIM^mut^ affected by HSJ1a and its mutants. The cell lysates as shown in (A) were subjected to IP with anti-HA antibody and the resulting precipitates were subjected to IB analysis with anti-Ub antibody (upper panel) or anti-HA antibody (lower panel). The control lane represents the background of immunoblotting by using the HA antibody (without cell lysates).
(PDF)

Figure S4 Hsp70 and CHIP regulate the degradation of Atx3. (A) HA-Atx3 and different amount of HA-HSP70 (0, 0.05, 0.1, 0.2, 0.4, 2 µg of DNA) were co-transfected into HEK 293T cells. After 48 hrs, the cell lysates were subjected to immunoblotting with indicated antibody. (B) As (A), with Myc-CHIP (0, 0.5, 1, 2 µg of DNA). (C) Equal amount of HA-tagged Atx3 and Myc-CHIP were co-transfected to HEK 293T cells. After 36 hrs, the cells were treated with 20 µM MG132 for 10 h, and then the cell lysates were subjected to immunoblotting with anti-Atx3 antibody.
(PDF)

Figure S5 Interaction between HSP90 and HSJ1a. (A) Co-IP experiment for interaction of HSJ1a with HSP90. The cell lysates with indicated proteins were subjected to co-IP with anti-His antibody and the resulting precipitates were subjected to immunoblotting with anti-HA (HSP90) and anti-Myc (HSJ1a) antibodies. (B) As (A), with anti-HA antibody for immunoprecipitation, and anti-Myc (HSj1a) and anti-HA (HSP90) antibodies for immunoblotting.
(PDF)

Figure S6 Co-localization of the inclusion bodies formed by polyQ-expanded Atx3 with endogenous HSP70 or HSJ1a. HEK 293T cells were transiently transfected with the vector expressing HA-Atx3_71Q_. After 48 hrs, the cells were subjected to immuno-fluorescence staining with anti-HSP70 (red) or anti-HSJ1a (red) and anti-HA (green) antibodies. Nuclei are stained with Hoechst (blue). Scale bar = 20 µm.
(PDF)

Figure S7 Effects of HSJ1a and its mutants on the protein levels of polyQ-expanded Atx3. (A) Effects of HSJ1a and its fragments on the protein levels of Atx371Q. HA-tagged Atx371Q were co-transfected with Myc-HSJ1a or its fragments in HEK 293T cells. About 48 hrs after transfection, the cell lysates were subjected to immunoblotting with anti-HA, anti-Myc and anti-actin antibodies. Atx3_71Q_, polyQ-expanded Atx3 with 71 Gln residues. (B) Effects of HSJ1a and its mutants on the Atx3_71Q_ levels in a dose-dependent manner. HA-Atx3_71Q_ and different amounts of Myc-HSJ1a or its mutants were co-transfected into HEK 293T cells. The amounts of plasmid DNA are 0, 0.5 and 2 µg, respectively. (C) Immuno-fluorescence imaging of the cellular protein levels of Atx3_71Q_ regulated by HSJ1a and its mutants. Atx371Q is stained with anti-HA antibody (green), HSJ1a and its mutants are stained with anti-Myc antibody (red), and nuclei are stained with Hoechst (blue). Scale bar = 20 µm.
(PDF)

Figure S8 HSJ1a and its mutants exert no effect on the protein level of endogenous Atx3. Myc-tagged HSJ1a, HSJ1a-JD^mut^, HSJ1a-UIM^mut^ and empty vector were transfected into HEK 293T cells. About 48 hrs after transfection, the cell lysates were subjected to immunoblotting with anti-Atx3, anti-Myc and anti-actin antibodies.
(PDF)

Figure S9 Ubiquitination of Atx3 in an *in vitro* system. (A) The ubiquitination of Atx3 was performed in a reaction mixture of UbE1, UbcH5C, Ub, ATP, and the HEK 293T cell lysates containing GST, GST-HSJ1a or its mutants. Atx3 and its ubiquitinated forms were detected by immunoblotting with an anti-Atx3 antibody. (B) The ubiquitination of Atx3 was performed in a reaction mixture of purified proteins as indicated. The reaction products were detected as in (A). The arrows denote the bands of Atx3 and HSJ1a without ubiquitination.
(PDF)
