# Supplementary material for: Co-Chaperone HSJ1a Dually Regulates the Proteasomal Degradation of Ataxin-3
Source: PLoS One. 2011 May 19;6(5):e19763. doi: 10.1371/journal.pone.0019763 (PMC3098244; doi:10.1371/journal.pone.0019763)
Supplement: Figure S1 — Effects of HSJ1a and its fragments on the mRNA levels of Atx3. (A) Sequences and domain architecture of HSJ1a and its fragments and mutants. (B) Quantitative real-time PCR analysis of Atx3 mRNA levels in HEK 293T cells transiently transfected with the indicated plasmids as shown. Shown are means ± S.D. (n = 3). JD, J-domain fragment; ΔJD, J-domain deleted fragment. (PDF) [file pone.0019763.s001.pdf]

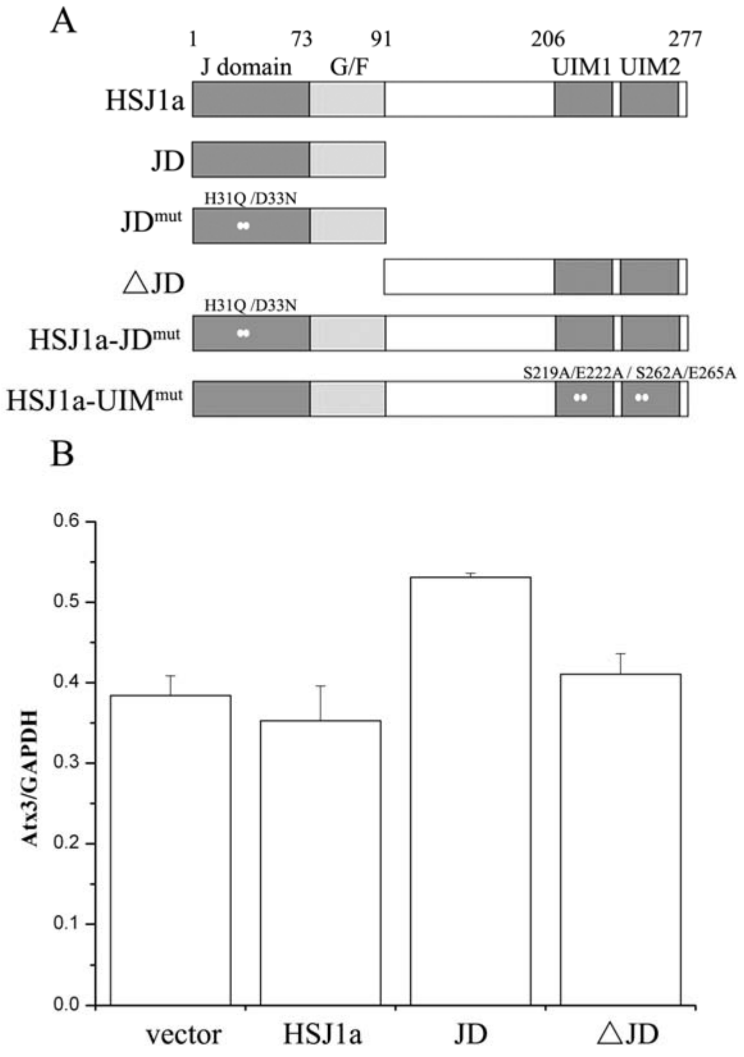

Figure S1 Effects of HSJ1a and its fragments on the mRNA levels of Atx3. (A) Sequences and domain architecture of HSJ1a and its fragments and mutants. (B) Quantitative real-time PCR analysis of Atx3 mRNA levels in HEK 293T cells transiently transfected with the indicated plasmids as shown. Shown are means ± S.D. (n =3). JD, J-domain fragment; ΔJD, J-domain deleted fragment.
