# Supplementary material for: Co-Chaperone HSJ1a Dually Regulates the Proteasomal Degradation of Ataxin-3
Source: PLoS One. 2011 May 19;6(5):e19763. doi: 10.1371/journal.pone.0019763 (PMC3098244; doi:10.1371/journal.pone.0019763)
Supplement: Figure S2 — Interactions between the UIM12 domain of HSJ1a and different diUb chains. (A) ITC experiment for the interaction of GB1-UIM12 with K48-diUb. The concentration of diUb is 100 µM and that of the GB1-UIM12 stock is 2 mM. The data are fitted with the one-site binding model. The GB1 fusion was used for purification and quantification. (B) As (A), with K63-diUb. (C) NMR titration of GB1-UIM12 with K48-diUb. Shown is the overlay of the 1H-15N HSQC spectra of 15N-labeled GB1-UIM12 before (green) and after (red) addition of 1 equiv. of K48-diUb. (D) As (C), with K63-diUb. Shown is the overlay of the 1H-15N HSQC spectra of 15N-labeled GB1-UIM12 before (blue) and after (red) addition of 1 equiv. of K63-diUb. (E) Plot of the relative peak heights against the residue number of GB1-UIM12 titrated with 1 equiv. of K48-diUb. All the peak heights were normalized except for prolines and unassigned residues. The diagram shows the peak heights for the residues from GB1 (yellow), UIM1 (blue), the linker (purple) and UIM2 (blue). (F) Co-IP experiment for interaction between HSJ1a or its UIM mutant and endogenous Ub chains. HEK 293T cells were transiently transfected with a vector expressing His-HSJ1a or His-HSJ1a-UIMmut. After 48 hrs, the cells were subjected to immunoprecipitation (IP) with anti-His and the resulting precipitates were subjected to immunoblotting (IB) with either anti-Ub or anti-His. The asterisks denote the bands from the heavy and light chains of IgG. (PDF) [file pone.0019763.s002.pdf]

Figure S2

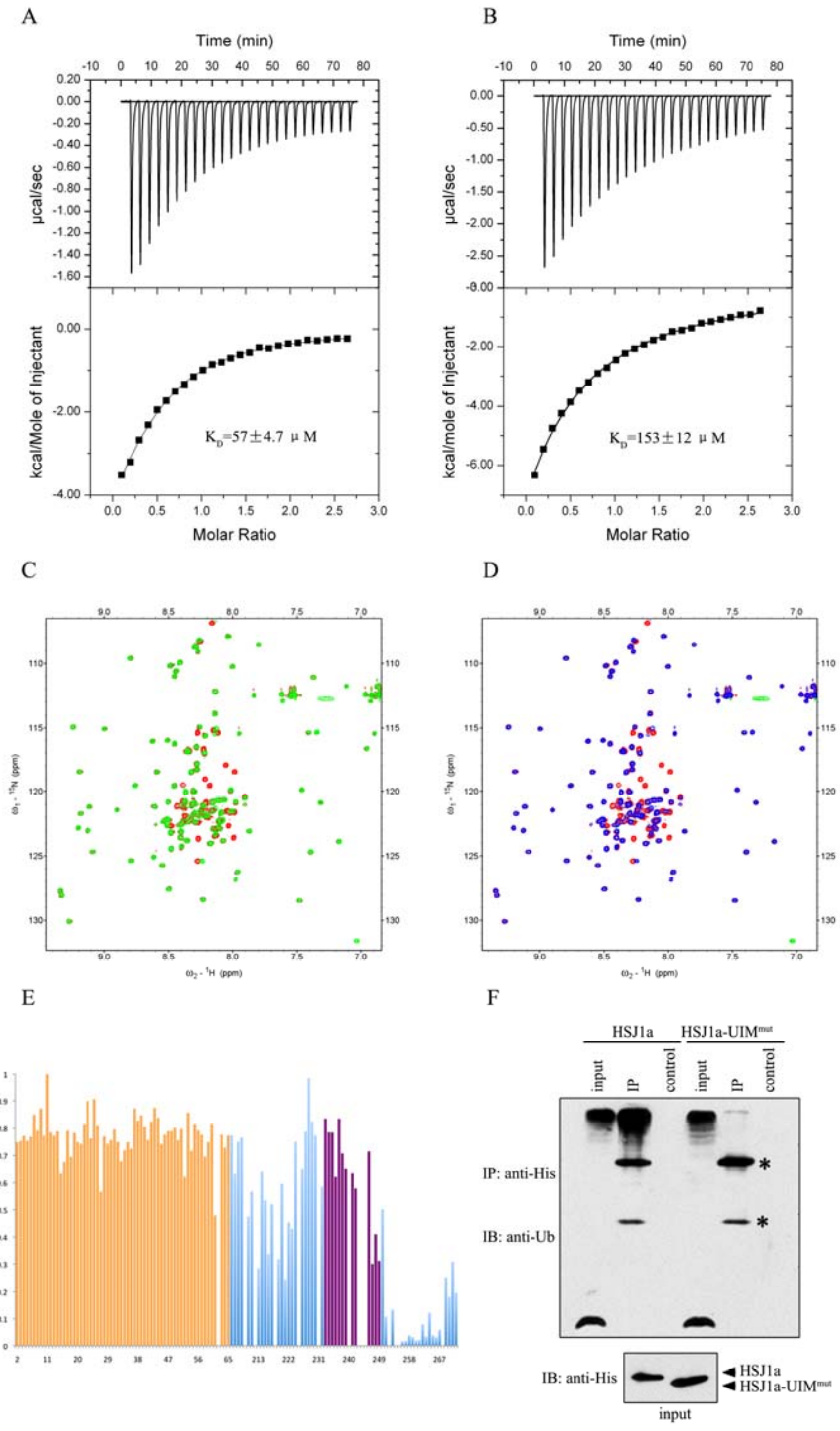

Figure S2 Interactions between the UIM12 domain of HSJ1a and different diUb chains. (A) ITC experiment for the interaction of GB1-UIM12 with K48-diUb. The concentration of diUb is 100 μM and that of the GB1-UIM12 stock is 2 mM. The data

are fitted with the one-site binding model. The GB1 fusion was used for purification and quantification. (B) As (A), with K63-diUb. (C) NMR titration of GB1-UIM12 with K48-diUb. Shown is the overlay of the ^{1}H-^{15}N HSQC spectra of ^{15}N-labeled GB1-UIM12 before (green) and after (red) addition of 1 equiv. of K48-diUb. (D) As (C), with K63-diUb. Shown is the overlay of the ^{1}H-^{15}N HSQC spectra of ^{15}N-labeled GB1-UIM12 before (blue) and after (red) addition of 1 equiv. of K63-diUb. (E) Plot of the relative peak heights against the residue number of GB1-UIM12 titrated with 1 equiv. of K48-diUb. All the peak heights were normalized except for prolines and unassigned residues. The diagram shows the peak heights for the residues from GB1 (yellow), UIM1 (blue), the linker (purple) and UIM2 (blue). (F) Co-IP experiment for interaction between HSJ1a or its UIM mutant and endogenous Ub chains. HEK 293T cells were transiently transfected with a vector expressing His-HSJ1a or His-HSJ1a-UIM^{mut}. After 48 hrs, the cells were subjected to immunoprecipitation (IP) with anti-His and the resulting precipitates were subjected to immunoblotting (IB) with either anti-Ub or anti-His. The asterisks denote the bands from the heavy and light chains of IgG.
