# Supplementary material for: Co-Chaperone HSJ1a Dually Regulates the Proteasomal Degradation of Ataxin-3
Source: PLoS One. 2011 May 19;6(5):e19763. doi: 10.1371/journal.pone.0019763 (PMC3098244; doi:10.1371/journal.pone.0019763)
Supplement: Figure S3 — HSJ1a exerts the similar effect on the UIM mutant form of Atx3. (A) Different effects of HSJ1a and its mutants on the protein levels of Atx3-UIMmut. HA-Atx3-UIMmut and Myc-HSJ1a or its mutants were co-transfected into HEK 293T cells. About 48 hrs after transfection, the cell lysates were subjected to immunoblotting with anti-HA and anti-Myc antibodies. (B) Ubiquitination of Atx3-UIMmut affected by HSJ1a and its mutants. The cell lysates as shown in (A) were subjected to IP with anti-HA antibody and the resulting precipitates were subjected to IB analysis with anti-Ub antibody (upper panel) or anti-HA antibody (lower panel). The control lane represents the background of immunoblotting by using the HA antibody (without cell lysates). (PDF) [file pone.0019763.s003.pdf]

Figure S3

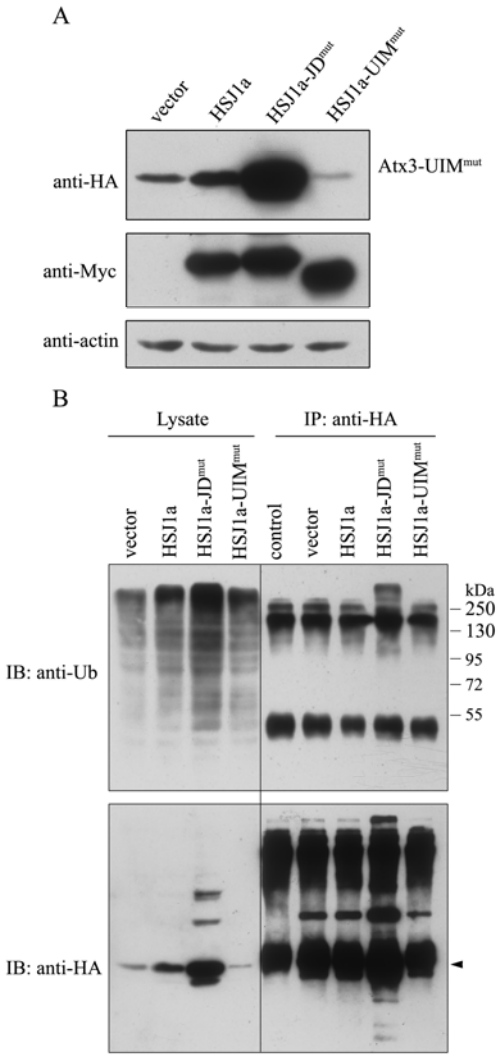

Figure S3 HSJ1a exerts the similar effect on the UIM mutant form of Atx3. (A) Different effects of HSJ1a and its mutants on the protein levels of Atx3-UIM^{mut}. HA-Atx3-UIM^{mut} and Myc-HSJ1a or its mutants were co-transfected into HEK 293T cells. About 48 hrs after transfection, the cell lysates were subjected to immunoblotting with anti-HA and anti-Myc antibodies. (B) Ubiquitination of Atx3-UIM^{mut} affected by HSJ1a and its mutants. The cell lysates as shown in (A) were subjected to IP with anti-HA antibody and the resulting precipitates were subjected to IB analysis with anti-Ub antibody (upper panel) or anti-HA antibody (lower panel). The control lane represents the background of immunoblotting by using the HA antibody (without cell lysates).
