# Supplementary material for: Co-Chaperone HSJ1a Dually Regulates the Proteasomal Degradation of Ataxin-3
Source: PLoS One. 2011 May 19;6(5):e19763. doi: 10.1371/journal.pone.0019763 (PMC3098244; doi:10.1371/journal.pone.0019763)
Supplement: Figure S4 — Hsp70 and CHIP regulate the degradation of Atx3. (A) HA-Atx3 and different amount of HA-HSP70 (0, 0.05, 0.1, 0.2, 0.4, 2 µg of DNA) were co-transfected into HEK 293T cells. After 48 hrs, the cell lysates were subjected to immunoblotting with indicated antibody. (B) As (A), with Myc-CHIP (0, 0.5, 1, 2 µg of DNA). (C) Equal amount of HA-tagged Atx3 and Myc-CHIP were co-transfected to HEK 293T cells. After 36 hrs, the cells were treated with 20 µM MG132 for 10 h, and then the cell lysates were subjected to immunoblotting with anti-Atx3 antibody. (PDF) [file pone.0019763.s004.pdf]

Figure S4

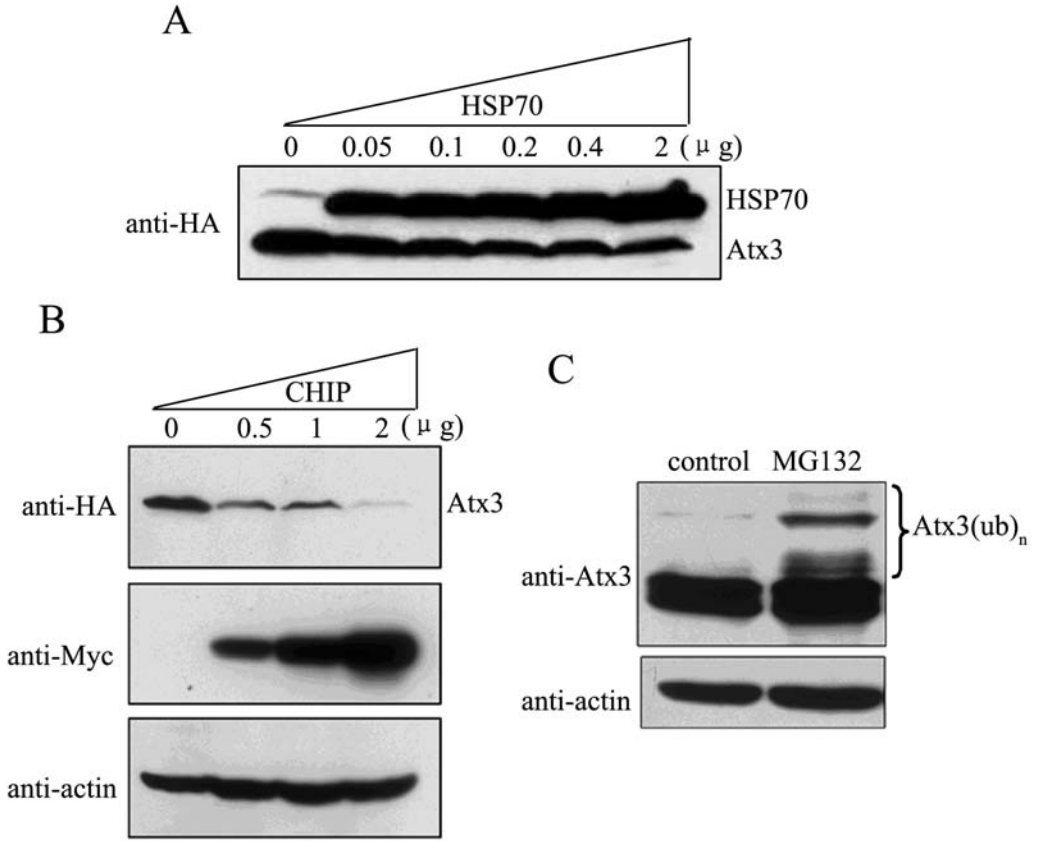

Figure S4 Hsp70 and CHIP regulate the degradation of Atx3. (A) HA-Atx3 and different amount of HA-HSP70 (0, 0.05, 0.1, 0.2, 0.4, 2 μg of DNA) were co-transfected into HEK 293T cells. After 48 hrs, the cell lysates were subjected to immunoblotting with indicated antibody. (B) As (A), with Myc-CHIP (0, 0.5, 1, 2 μg of DNA). (C) Equal amount of HA-tagged Atx3 and Myc-CHIP were co-transfected to HEK 293T cells. After 36 hrs, the cells were treated with 20 μM MG132 for 10h, and then the cell lysates were subjected to immunoblotting with anti-Atx3 antibody.
