# Supplementary material for: Co-Chaperone HSJ1a Dually Regulates the Proteasomal Degradation of Ataxin-3
Source: PLoS One. 2011 May 19;6(5):e19763. doi: 10.1371/journal.pone.0019763 (PMC3098244; doi:10.1371/journal.pone.0019763)
Supplement: Figure S5 — Interaction between HSP90 and HSJ1a. (A) Co-IP experiment for interaction of HSJ1a with HSP90. The cell lysates with indicated proteins were subjected to co-IP with anti-His antibody and the resulting precipitates were subjected to immunoblotting with anti-HA (HSP90) and anti-Myc (HSJ1a) antibodies. (B) As (A), with anti-HA antibody for immunoprecipitation, and anti-Myc (HSj1a) and anti-HA (HSP90) antibodies for immunoblotting. (PDF) [file pone.0019763.s005.pdf]

Figure S5

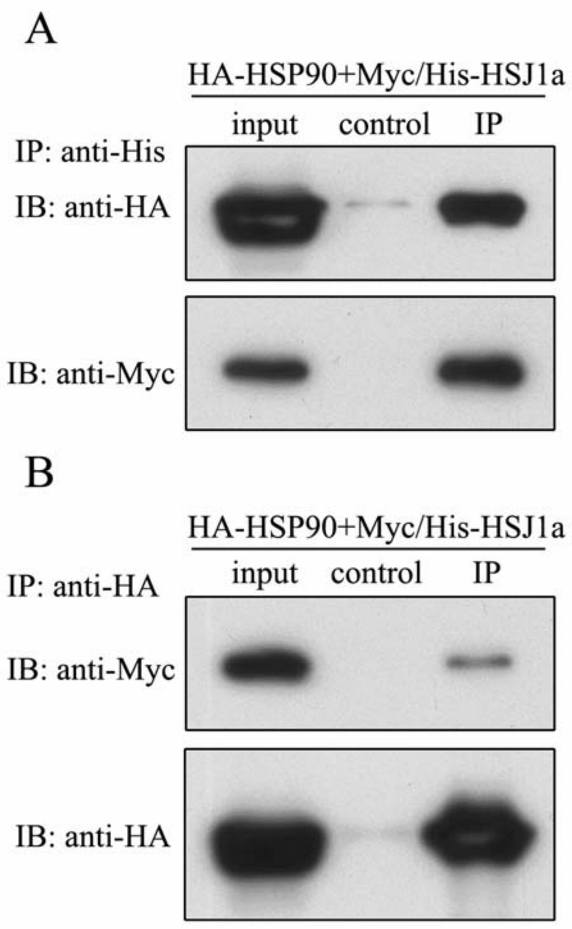

Figure S5 Interaction between HSP90 and HSJ1a. (A) Co-IP experiment for interaction of HSJ1a with HSP90. The cell lysates with indicated proteins were subjected to co-IP with anti-His antibody and the resulting precipitates were subjected to immunoblotting with anti-HA (HSP90) and anti-Myc (HSJ1a) antibodies. (B) As (A), with anti-HA antibody for immunoprecipitation, and anti-Myc (HSj1a) and anti-HA (HSP90) antibodies for immunoblotting.
