# Supplementary material for: Co-Chaperone HSJ1a Dually Regulates the Proteasomal Degradation of Ataxin-3
Source: PLoS One. 2011 May 19;6(5):e19763. doi: 10.1371/journal.pone.0019763 (PMC3098244; doi:10.1371/journal.pone.0019763)
Supplement: Figure S6 — Co-localization of the inclusion bodies formed by polyQ-expanded Atx3 with endogenous HSP70 or HSJ1a. HEK 293T cells were transiently transfected with the vector expressing HA-Atx371Q. After 48 hrs, the cells were subjected to immuno-fluorescence staining with anti-HSP70 (red) or anti-HSJ1a (red) and anti-HA (green) antibodies. Nuclei are stained with Hoechst (blue). Scale bar = 20 µm. (PDF) [file pone.0019763.s006.pdf]

Figure S6

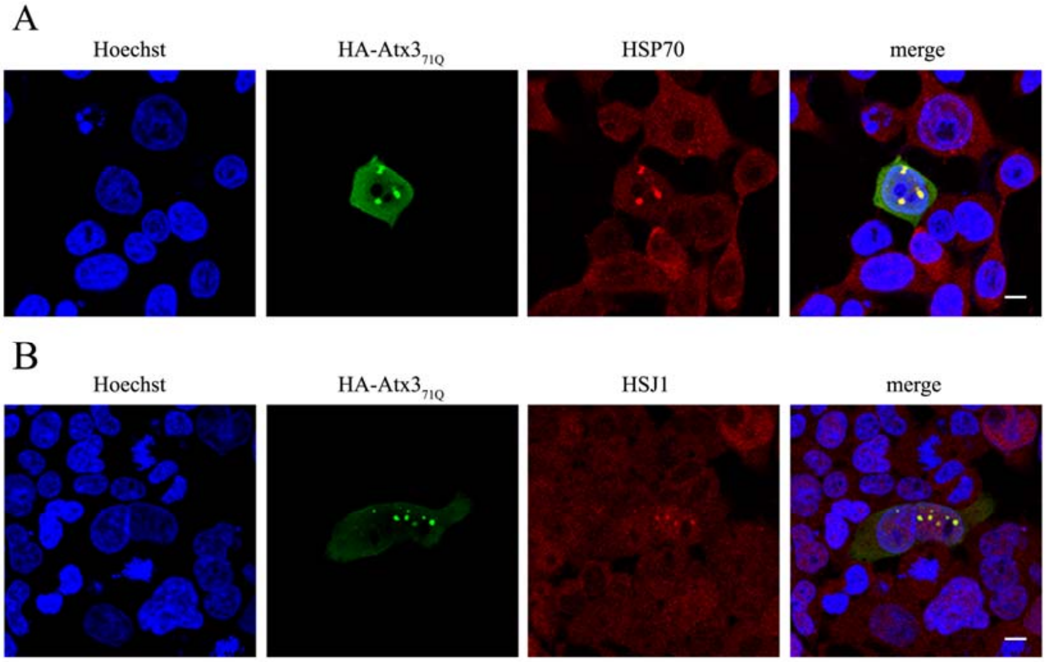

Figure S6 Co-localization of the inclusion bodies formed by polyQ-expanded Atx3 with endogenous HSP70 or HSJ1a. HEK 293T cells were transiently transfected with the vector expressing HA-Atx3$_{71Q}$. After 48 hrs, the cells were subjected to immuno-fluorescence staining with anti-HSP70 (red) or anti-HSJ1a (red) and anti-HA (green) antibodies. Nuclei are stained with Hoechst (blue). Scale bar = 20 µm.
