# Supplementary material for: Co-Chaperone HSJ1a Dually Regulates the Proteasomal Degradation of Ataxin-3
Source: PLoS One. 2011 May 19;6(5):e19763. doi: 10.1371/journal.pone.0019763 (PMC3098244; doi:10.1371/journal.pone.0019763)
Supplement: Figure S7 — Effects of HSJ1a and its mutants on the protein levels of polyQ-expanded Atx3. (A) Effects of HSJ1a and its fragments on the protein levels of Atx371Q. HA-tagged Atx371Q were co-transfected with Myc-HSJ1a or its fragments in HEK 293T cells. About 48 hrs after transfection, the cell lysates were subjected to immunoblotting with anti-HA, anti-Myc and anti-actin antibodies. Atx371Q, polyQ-expanded Atx3 with 71 Gln residues. (B) Effects of HSJ1a and its mutants on the Atx371Q levels in a dose-dependent manner. HA-Atx371Q and different amounts of Myc-HSJ1a or its mutants were co-transfected into HEK 293T cells. The amounts of plasmid DNA are 0, 0.5 and 2 µg, respectively. (C) Immuno-fluorescence imaging of the cellular protein levels of Atx371Q regulated by HSJ1a and its mutants. Atx371Q is stained with anti-HA antibody (green), HSJ1a and its mutants are stained with anti-Myc antibody (red), and nuclei are stained with Hoechst (blue). Scale bar = 20 µm. (PDF) [file pone.0019763.s007.pdf]

Figure S7

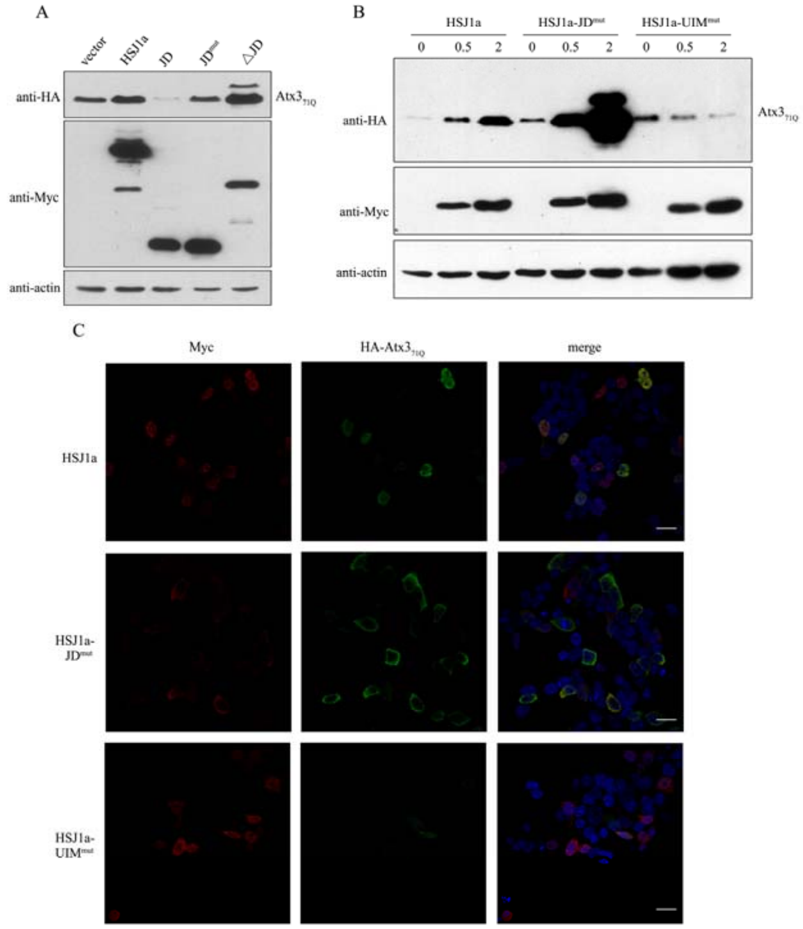

Figure S7 Effects of HSJ1a and its mutants on the protein levels of polyQ-expanded Atx3. (A) Effects of HSJ1a and its fragments on the protein levels of $Atx3_{71Q}$. HA-tagged $Atx3_{71Q}$ were co-transfected with Myc-HSJ1a or its fragments in HEK 293T cells. About 48 hrs after transfection, the cell lysates were subjected to immunoblotting with anti-HA, anti-Myc and anti-actin antibodies. $Atx3_{71Q}$, polyQ-expanded Atx3 with 71 Gln residues. (B) Effects of HSJ1a and its mutants on the $Atx3_{71Q}$ levels in a dose-dependent manner. HA-$Atx3_{71Q}$ and different amounts of Myc-HSJ1a or its mutants were co-transfected into HEK 293T cells. The amounts of plasmid DNA are 0, 0.5 and 2 µg, respectively. (C) Immuno-fluorescence imaging of the cellular protein levels of $Atx3_{71Q}$ regulated by HSJ1a and its mutants. $Atx3_{71Q}$ is stained with anti-HA antibody (green), HSJ1a and its mutants are stained with anti-Myc antibody (red), and nuclei are stained with Hoechst (blue). Scale bar = 20 µm.
