# Supplementary material for: Co-Chaperone HSJ1a Dually Regulates the Proteasomal Degradation of Ataxin-3
Source: PLoS One. 2011 May 19;6(5):e19763. doi: 10.1371/journal.pone.0019763 (PMC3098244; doi:10.1371/journal.pone.0019763)
Supplement: Figure S8 — HSJ1a and its mutants exert no effect on the protein level of endogenous Atx3. Myc-tagged HSJ1a, HSJ1a-JDmut, HSJ1a-UIMmut and empty vector were transfected into HEK 293T cells. About 48 hrs after transfection, the cell lysates were subjected to immunoblotting with anti-Atx3, anti-Myc and anti-actin antibodies. (PDF) [file pone.0019763.s008.pdf]

Figure S8

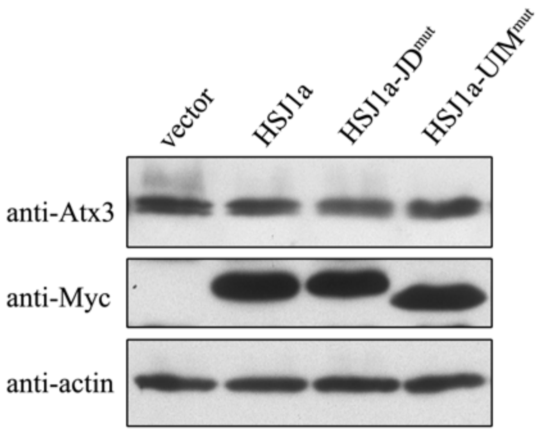

Figure S8 HSJ1a and its mutants exert no effect on the protein level of endogenous Atx3. Myc-tagged HSJ1a, HSJ1a-JD^{mut}, HSJ1a-UIM^{mut} and empty vector were transfected into HEK 293T cells. About 48 hrs after transfection, the cell lysates were subjected to immunoblotting with anti-Atx3, anti-Myc and anti-actin antibodies.
