# Supplementary material for: Co-Chaperone HSJ1a Dually Regulates the Proteasomal Degradation of Ataxin-3
Source: PLoS One. 2011 May 19;6(5):e19763. doi: 10.1371/journal.pone.0019763 (PMC3098244; doi:10.1371/journal.pone.0019763)
Supplement: Figure S9 — Ubiquitination of Atx3 in an in vitro system. (A) The ubiquitination of Atx3 was performed in a reaction mixture of UbE1, UbcH5C, Ub, ATP, and the HEK 293T cell lysates containing GST, GST-HSJ1a or its mutants. Atx3 and its ubiquitinated forms were detected by immunoblotting with an anti-Atx3 antibody. (B) The ubiquitination of Atx3 was performed in a reaction mixture of purified proteins as indicated. The reaction products were detected as in (A). The arrows denote the bands of Atx3 and HSJ1a without ubiquitination. (PDF) [file pone.0019763.s009.pdf]

Figure S9

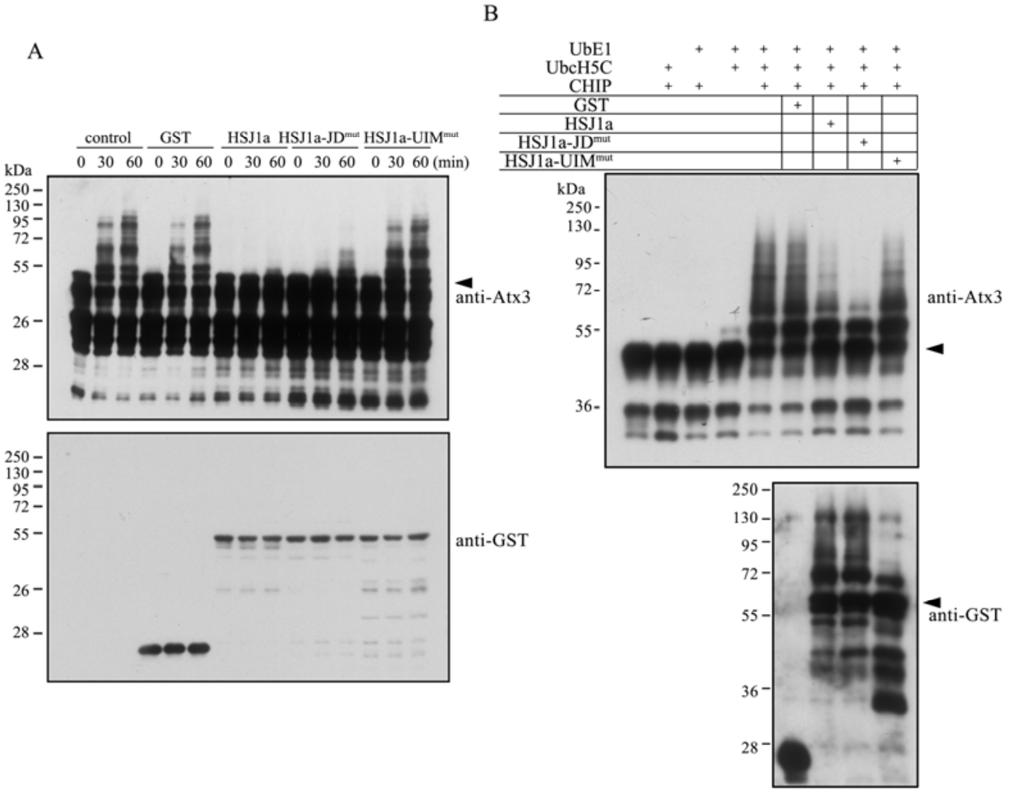

Figure S9 Ubiquitination of Atx3 in an *in vitro* system. (A) The ubiquitination of Atx3 was performed in a reaction mixture of UbE1, UbcH5C, Ub, ATP, and the HEK 293T cell lysates containing GST, GST-HSJ1a or its mutants. Atx3 and its ubiquitinated forms were detected by immunoblotting with an anti-Atx3 antibody. (B) The ubiquitination of Atx3 was performed in a reaction mixture of purified proteins as indicated. The reaction products were detected as in (A). The arrows denote the bands of Atx3 and HSJ1a without ubiquitination.
